# Structural and functional insights into the modulation of the activity of a flax cytokinin oxidase by flax rust effector AvrL567‐A

**DOI:** 10.1111/mpp.12749

**Published:** 2018-11-15

**Authors:** Li Wan, Markus Koeck, Simon J. Williams, Anthony R. Ashton, Gregory J. Lawrence, Hitoshi Sakakibara, Mikiko Kojima, Christine Böttcher, Daniel J. Ericsson, Adrienne R. Hardham, David A. Jones, Jeffrey G. Ellis, Bostjan Kobe, Peter N. Dodds

**Affiliations:** ^1^ School of Chemistry and Molecular Biosciences, Australian Infectious Diseases Research Centre and Institute for Molecular Bioscience University of Queensland Brisbane QLD 4072 Australia; ^2^ Department of Biology University of North Carolina Chapel Hill North Carolina 27599‐3280 USA; ^3^ Commonwealth Scientific and Industrial Research Organisation Agriculture and Food Canberra ACT 2601 Australia; ^4^ Division of Plant Sciences, Research School of Biology Australian National University Canberra ACT 2601 Australia; ^5^ RIKEN Center for Sustainable Resource Science Yokohama Kanagawa 230‐0045 Japan; ^6^ Commonwealth Scientific and Industrial Research Organisation Agriculture and Food Adelaide SA 5064 Australia; ^7^ Australian Synchrotron Macromolecular Crystallography Clayton Victoria 3168 Australia

**Keywords:** crystal structure, cytokinin, cytokinin oxidase, flax rust (*Melampsora lini*) effector, pathogen virulence

## Abstract

During infection, plant pathogens secrete effector proteins to facilitate colonization. In comparison with our knowledge of bacterial effectors, the current understanding of how fungal effectors function is limited. In this study, we show that the effector AvrL567‐A from the flax rust fungus *Melampsora lini* interacts with a flax cytosolic cytokinin oxidase, LuCKX1.1, using both yeast two‐hybrid and *in planta* bimolecular fluorescence assays. Purified LuCKX1.1 protein shows catalytic activity against both N6‐(Δ2‐isopentenyl)‐adenine (2iP) and *trans*‐zeatin (tZ) substrates. Incubation of LuCKX1.1 with AvrL567‐A results in increased catalytic activity against both substrates. The crystal structure of LuCKX1.1 and docking studies with AvrL567‐A indicate that the AvrL567 binding site involves a flexible surface‐exposed region that surrounds the cytokinin substrate access site, which may explain its effect in modulating LuCKX1.1 activity. Expression of AvrL567‐A in transgenic flax plants gave rise to an epinastic leaf phenotype consistent with hormonal effects, although no difference in overall cytokinin levels was observed. We propose that, during infection, plant pathogens may differentially modify the levels of extracellular and intracellular cytokinins.

## Introduction

The outcomes of plant–microbe interactions are largely determined by the effector repertoire secreted by pathogenic microbes during infection (Dou and Zhou, 2012; Toruno *et al.*, 2016). Pathogen effectors can act either in the plant apoplastic space or may be internalized into plant cells. Intracellular effectors often function to interfere with pathogen‐associated molecular pattern (PAMP)‐triggered immunity (PTI), which involves the recognition of PAMPs by plant cell surface receptors, known as pattern recognition receptors. In addition, some effectors that function in plant cells, called avirulence (Avr) proteins, are recognized by plant resistance proteins of the NLR (nucleotide‐binding leucine‐rich repeat receptor) class, leading to effector‐triggered immunity (ETI) (Dodds and Rathjen, 2010). Investigations of the signalling pathways hijacked by pathogen effectors for the enhancement of virulence are critical for an understanding of pathogen virulence mechanisms and plant immunity. Bacterial pathogens utilize type III secretion systems to deliver effectors into plant cells, and many studies have shown that these effectors subvert plant immunity and promote pathogen survival by interfering with PTI, ETI, proteasome‐dependent protein degradation, phytohormone signalling, cytoskeleton assembly, vesicle transport or gene expression (Buttner, 2016; Toruno *et al.*, 2016).

Unlike bacterial effectors, a limited number of cytosolic fungal effectors have been functionally characterized (Selin *et al.*, 2016). For example, *Ustilago maydis*, a hemibiotrophic fungal pathogen, secretes the effector Cmu1, affecting salicylic acid (SA) biosynthesis in plants to inhibit SA‐mediated immunity (Djamei *et al.*, 2011). In contrast with the relatively small set of effectors produced by individual species of bacterial pathogens, whole‐genome sequencing of biotrophic and hemibiotrophic fungi has identified hundreds of potential effectors (Dean *et al.*, 2005; Duplessis *et al.*, 2011; Kamper *et al.*, 2006; Miller *et al.*, 2018; Nemri *et al.*, 2014; Pedersen *et al.*, 2012; Plissonneau *et al.*, 2017; Schwessinger *et al.*, 2018; Spanu *et al.*, 2010; Yoshida *et al.*, 2009). Functional characterization of fungal effectors is challenging, mainly because fungal effector mutants often display no associated phenotype, either as a result of functional redundancy or of difficulty in accurately measuring small changes in phenotype (Selin *et al.*, 2016).

The biotrophic fungus *Melampsora lini* is the causal agent of flax rust disease on its host plant flax (*Linum usitatissimum*). During infection, flax rust develops feeding structures, called haustoria, which penetrate the cell wall and invaginate the plasma membrane of flax cells for both nutrient uptake and effector delivery (Garnica *et al.*, 2014). Several families of effectors have been identified from flax rust, including AvrL567, AvrM, AvrP, AvrP4, AvrL2 and AvrM14 (Anderson *et al.*, 2016; Nemri *et al.*, 2014). These effector proteins contain N‐terminal signal peptides that direct their secretion into the extrahaustorial matrix (Catanzariti *et al.*, 2006; Dodds *et al.*, 2004). The crystal structures of AvrL567, AvrM and AvrP have been reported and surface‐exposed residues that are important for recognition by corresponding flax NLR proteins have been identified (Ve *et al.*, 2013; Wang *et al.*, 2007; Zhang *et al.*, 2017). Despite a knowledge of their three‐dimensional structures, the virulence functions of these flax rust effectors have not yet been defined.

In this study, the flax cytosolic cytokinin oxidase/dehydrogenase LuCKX1.1, an enzyme involved in the irreversible degradation of hormones in the cytokinin family (Avalbaev *et al.*, 2012), was identified as a potential virulence target of the flax rust effector AvrL567‐A. Cytokinins are responsible for the regulation of cell division and plant development, and have also been implicated in plant immunity (Zurcher and Muller, 2016). We investigated the interactions between AvrL567‐A and LuCKX1.1, and demonstrated that AvrL567‐A increases the enzymatic activity of LuCKX1.1 *in vitro*. The crystal structure of LuCKX1.1 was determined, providing insights into the molecular basis of the LuCKX1–AvrL567‐A interaction and the action of the effector on LuCKX1.1 enzymatic activity. Flax plants expressing an AvrL567‐A transgene showed leaf epinasty, suggesting a possible hormonal imbalance, but no difference in overall cytokinin levels was detected relative to non‐transgenic flax plants.

## Results

### Flax cytokinin oxidase LuCKX1 interacts with the flax rust effector AvrL567‐A in yeast

**Figure 1:**
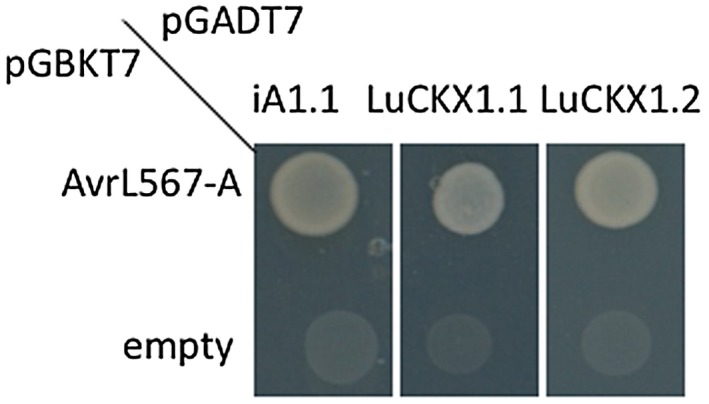
AvrL567‐A and LuCKX1.1 interact in yeast. Growth of yeast cells expressing AvrL567‐A in pGBKT7 or empty pGBKT7 in combination with iA1.1, LuCKX1.1 or LuCKX1.2 in pGADT7 on selective medium lacking tryptophan, leucine and histidine (–HTL). [Colour figure can be viewed at wileyonlinelibrary.com]

To identify potential virulence targets of the secreted flax rust effector AvrL567‐A, a yeast two‐hybrid (Y2H) screen was conducted with AvrL567‐A as bait and a cDNA library from infected leaves of a susceptible flax genotype as prey. One interacting clone iA1.1 (Fig. 1) encoded a polypeptide closely related to part of the *Arabidopsis thaliana* cytosolic cytokinin oxidase 7 (AtCKX7), suggesting that iA1.1 may be derived from a fragment of a flax cytokinin oxidase. We subsequently isolated two closely related full‐length cDNA clones from flax using the iA1.1 probe, which were termed *Linum usitatissimum* cytokinin oxidase 1.1 and 1.2 (*LuCKX1.1* and *LuCKX1.2*), respectively. AvrL567‐A interacted with both full‐length LuCKX1.1 and LuCKX1.2 in yeast (Fig. 1). The *LuCKX1.1* and *LuCKX1.2* genes are likely to be homeologues in the autotetraploid flax genome. Plants contain multiple enzymes of the cytokinin oxidase/dehydrogenase (CKX) family. For example, *A. thaliana* possesses seven genes (*AtCKX1* to *AtCKX7*) with different expression patterns and encoding proteins, and different extracellular and subcellular locations (Werner *et al.*, 2006). We identified 16 CKX‐related genes in the flax genome assembly (Cloutier *et al*., 2014) and phylogenetic analysis (Fig. S1, see Supporting Information) indicated that LuCKX1.1 and LuCKX1.2 were most closely related to AtCKX7 (63% amino acid identity), the only predicted cytosolic CKX protein in Arabidopsis (Kollmer *et al.*, 2014).

### LuCKX1.1 localizes to the cytosol and interacts with AvrL567‐A *in planta*

**Figure 2:**
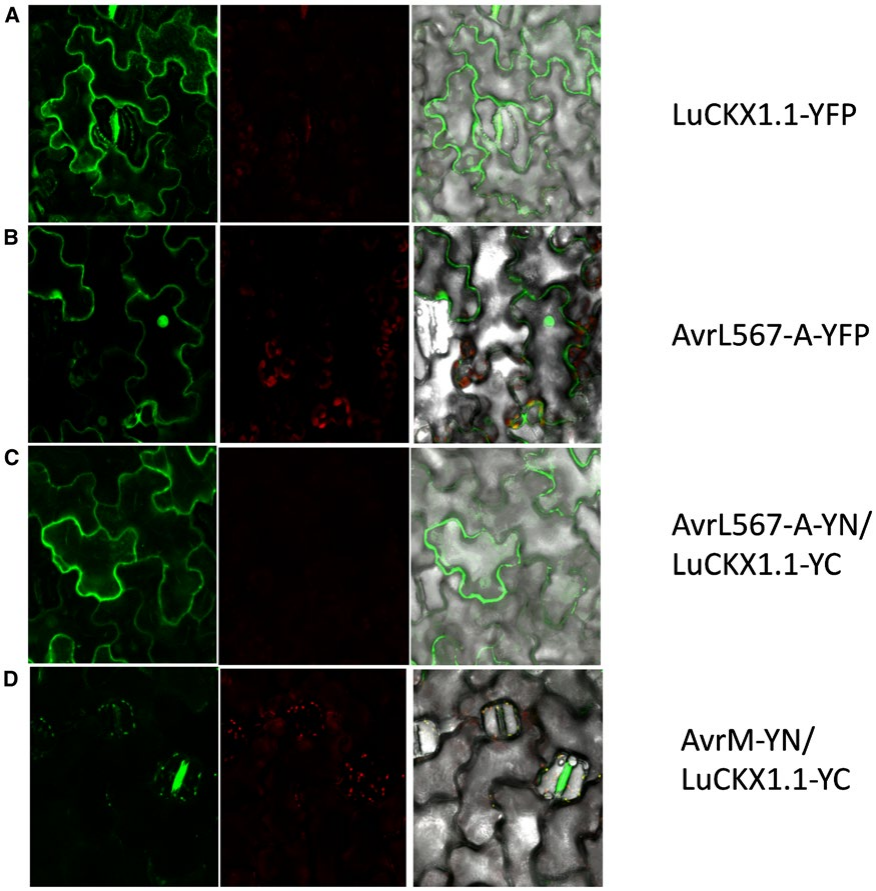
Subcellular localization of LuCKX1.1 and AvrL567‐A and bimolecular fluorescence complementation (BiFC) assays with LuCKX1.1‐YC and AvrL567‐YN or AvrM‐YN. Confocal images of flax leaves transiently expressing LuCKX1.1‐YFP (A), AvrL567‐A‐YFP (B), LuCKX1.1‐YC and AvrL567‐YN (C), and LuCKX1.1‐YC and AvrM‐YN (D). Images in (A) and (B) were taken at 4 days post‐inoculation (dpi) and in (C) and (D) at 3 dpi. Left panels, YFP signal; middle panels, chloroplast fluorescence channel; right panels, overlay of YFP, chloroplast and bright field channels. YFP, yellow fluorescent protein. [Colour figure can be viewed at wileyonlinelibrary.com]

**Figure 3:**
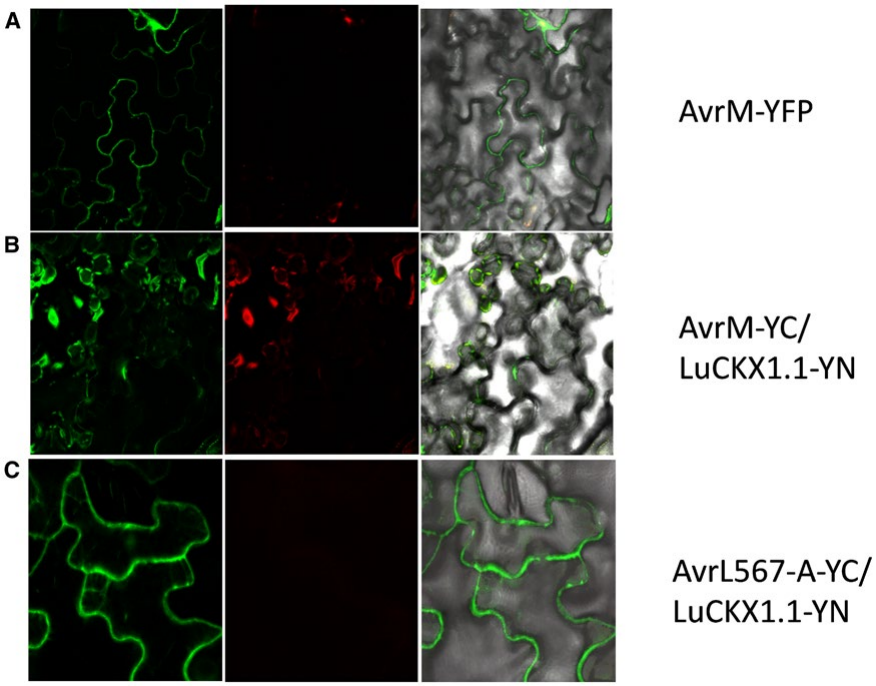
Subcellular localization of AvrM and bimolecular fluorescence complementation (BiFC) assays with LuCKX1.1‐YN and AvrL567‐YC or AvrM‐YC. Confocal images of flax leaves transiently expressing AvrM‐YFP (A), AvrM‐YC and LuCKX1.1‐YN (B), and AvrL567‐A‐YC and LuCKX1.1‐YN (C). Images in (A) were taken at 4 days post‐inoculation (dpi) and in (B) and (C) at 3 dpi. Left panels, YFP signal; middle panels, chloroplast fluorescence channel; right panels, overlay of YFP, chloroplast and bright field images. YFP, yellow fluorescent protein. [Colour figure can be viewed at wileyonlinelibrary.com]

When expressed under the control of the 35S promoter in flax leaves, a LuCKX1.1‐YFP (yellow fluorescent protein) fusion protein localized to the cytosol, but was excluded from the nucleus, a distribution consistent with its large size, the absence of a secretory signal and its close relationship to cytosolic Arabidopsis AtCKX7 (Fig. 2A). AvrL567‐A‐YFP localized to the nucleus and the cytoplasm (Fig. 2B), as observed previously (Rafiqi *et al.*, 2010). To test the interaction between AvrL567‐A and LuCKX1.1 *in planta*, we performed a bimolecular fluorescence complementation (BiFC) assay (Walter *et al.*, 2004). Co‐expression of AvrL567‐A fused to the N‐terminal fragment of YFP (YN) and LuCKX1.1 fused to the C‐terminal fragment of YFP (YC) resulted in the reconstitution of YFP fluorescence in the cytosol (Fig. 2C). Co‐expression of LuCKX1.1‐YC with the unrelated flax rust effector AvrM fused to YN did not give rise to any YFP fluorescence (Fig. 2D). Interchanging the YN and YC tags in the BiFC experiments did not alter the results obtained for LuCKX1.1–AvrL567‐A and LuCKX1.1–AvrM interactions (Fig. 3). The expression of all fusion proteins *in planta* was confirmed by protein gel blots (Fig. S2, see Supporting Information).

### AvrL567‐A enhances the enzymatic activity of LuCKX1.1 *in vitro*

**Figure 4:**
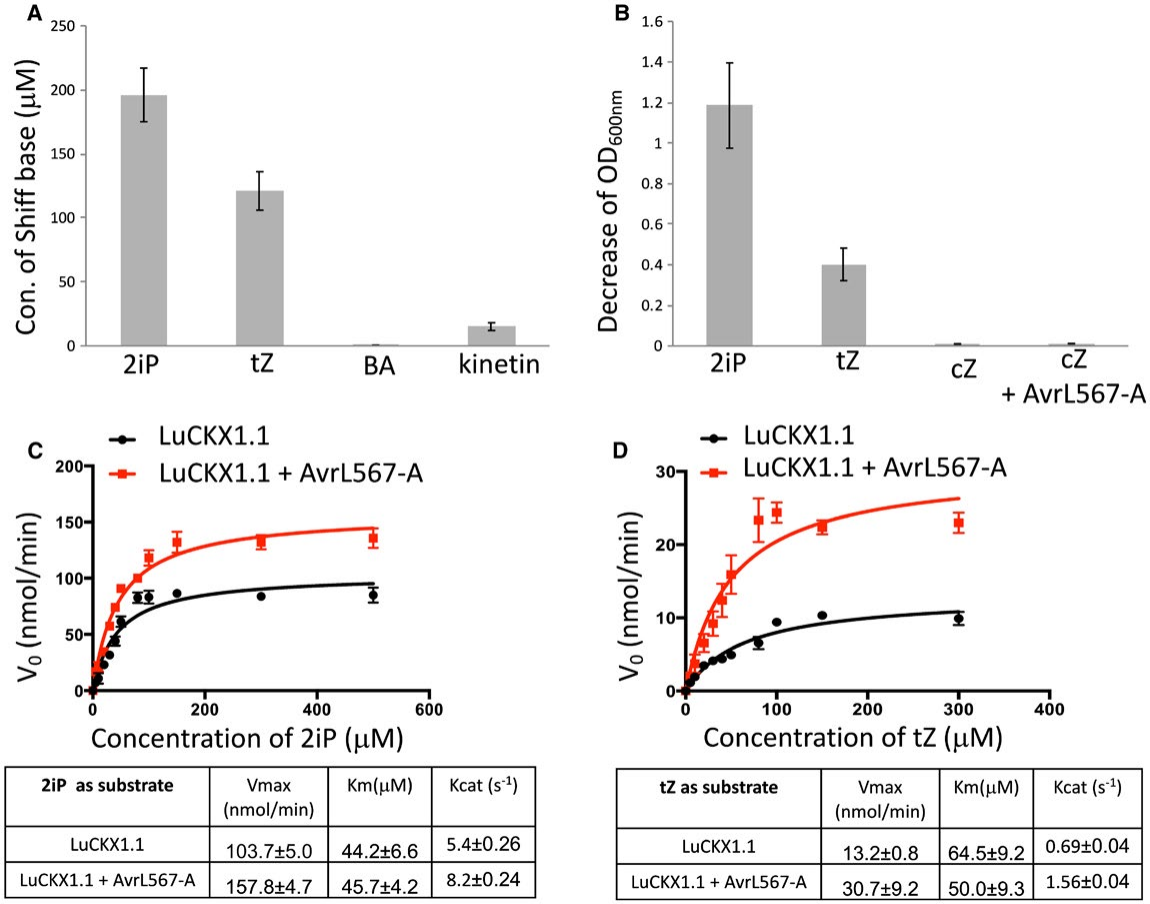
AvrL567‐A enhances LuCKX1.1 activity *in vitro*. (A) The concentrations of Schiff bases were measured in an endpoint assay to indicate the conversions of 2‐isopentenyladenine (2iP), *trans*‐zeatin (tZ), N6‐benzyladenine (BA) and N6‐furfuryladenine (kinetin) by LuCKX1.1. (B) The bleaching of 2,6‐dichlorophenolindophenol (DCPIP) due to reduction, as an electron acceptor in the LuCKX1.1‐mediated cytokinin oxidation, was measured in this assay to indicate the oxidative conversions of 2iP, tZ, *cis*‐zeatin (cZ) and cZ in the presence of AvrL567‐A. (C, D) Kinetic assays on LuCKX1.1 represented as Michaelis–Menten plots. The concentrations of substrates 2iP (C) and tZ (D) were plotted against the initial velocity of the enzymatic reaction (*V*_0_). Error bars represent standard errors from three independent measurements. Black and red lines represent the non‐linear fits for these plots from which the enzymatic parameters were calculated. The figure was prepared using GraphPad Prism version 5.0. [Colour figure can be viewed at wileyonlinelibrary.com]

To examine the enzymatic activity of LuCKX1.1 and whether the AvrL567‐A interaction influenced this activity, the AvrL567‐A (residues 24–150) and LuCKX1.1 (residues 44–534) proteins were expressed and purified as described previously (Wan *et al.*, 2013; Wang *et al.*, 2007). Endpoint assays (Frebort *et al.*, 2002) were performed to investigate the substrate specificities of LuCKX1.1. Four common types of cytokinin, 2‐isopentenyladenine (2iP), *trans*‐zeatin (tZ), N6‐benzyladenine (BA) and N6‐furfuryladenine (kinetin), were used as substrates. The results indicated that LuCKX1.1 preferentially catalyses the oxidative cleavage of 2iP and tZ over the aromatic cytokinin types BA or kinetin (Fig. 4A). As the endpoint assay measures Schiff bases (Frebort *et al.*, 2002), it is not suitable for *cis*‐zeatin (cZ). An assay based on the measurement of the bleaching of the electron acceptor 2,6‐dichlorophenolindophenol (DCPIP) (Laskey *et al.*, 2003) was therefore employed to examine the ability of LuCKX1.1 to cleave cZ. The results confirmed that LuCKX1.1 can efficiently cleave 2iP and tZ, but showed that it has negligible activity towards cZ in the absence or presence of AvrL567‐A (Fig. 4B).

To obtain kinetic information on LuCKX1.1 enzymatic activity, a modified DCPIP assay was conducted in which the initial reaction rates were calculated against a range of cytokinin concentrations (Fig. 4C,D). LuCKX1.1 showed similar *K*_m_ values of 44 and 64 µM for the two preferred substrates 2iP and tZ, respectively, but a higher catalytic rate (*K*_cat_) for 2iP (5.4 s^−1^ vs. 0.69 s^−1^). Significantly, in the presence of AvrL567, the turnover rate of LuCKX1.1 towards 2iP and tZ increased by about 50% and 130%, respectively, suggesting that this rust effector may act to affect intracellular cytokinin levels. Despite the strong effect on enzymatic activity, we did not detect a physical interaction between purified AvrL567‐A and LuCKX1.1 proteins by glutathione S‐transferase (GST) pull‐down or isothermal titration calorimetry, suggesting that the interaction *in vitro* might be weak or transient.

### Crystal structure of LuCKX1.1

To obtain insights into the molecular basis of the AvrL567‐A–LuCKX1.1 interaction, the flax LuCKX1.1 protein (residues 44–534) was crystallized as described previously (Wan *et al.*, 2013). The structure was solved by molecular replacement using Phaser (McCoy *et al.*, 2007) with AtCKX7 (PDB ID: 2EXR) as the template. The final refined model of LuCKX1.1 contains residues 44–532. Refinement statistics are summarized in Table S1 (see Supporting Information).

**Figure 5:**
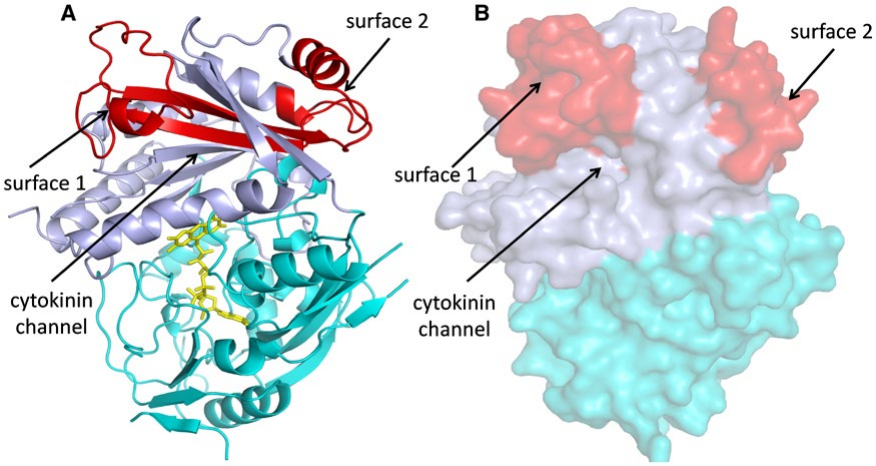
Structure of LuCKX1.1 and the iA1.1 region. The flavin adenine dinucleotide (FAD)‐binding and substrate‐binding domains are shown in cyan and light blue, respectively. The identified minimal binding region in iA1.1 is coloured red. Surface 1, surface 2 and the cytokinin channel are indicated by black arrows. (A) Cartoon representation of LuCKX1.1 structure. The FAD molecule is shown in stick representation in yellow. (B) The LuCKX1.1 structure is shown in surface representation. [Colour figure can be viewed at wileyonlinelibrary.com]

A search of the Protein Data Bank (PDB) with the program DALI (Holm and Rosenstrom, 2010) showed that the most similar structure is that of AtCKX7 with a Cα root‐mean‐square distance (rmsd) of 1.5 Å for 491 equivalent Cα atoms. The structure of LuCKX1.1 exhibits a two‐domain topology consisting of a flavin adenine dinucleotide (FAD)‐binding domain and a substrate‐binding domain (Fig. 5), which is similar to the known structures of AtCKX7, ZmCKX1, ZmCKX2, ZmCKX4a and ZmCKX4b (Bae *et al.*, 2008; Kopecny *et al.*, 2016; Malito *et al.*, 2004). LuCKX1.1 contains the FAD co‐factor covalently linked to histidine‐104 (His104) via the 8‐methyl group of the flavin ring in LuCKX1.1. The FAD‐binding domain (residues 44–243 and 478–532) comprises one mixed β‐sheet, one antiparallel β‐sheet and flanking α‐helices. The other domain (residues 244–477) contains an antiparallel β‐sheet surrounded by α‐helices. The corresponding domain in the structures of ZmCKX1 and ZmCKX2 complexed with different cytokinins is involved in substrate binding (Kopecny *et al.*, 2016; Malito *et al.*, 2004). The residues important for substrate binding identified in the active sites of ZmCKX1 and ZmCKX2 are largely conserved in LuCKX1.1, including aspartic acid‐168 (Asp168), glutamic acid‐281 (Glu281), valine‐365 (Val365), glutamic acid‐369 (Glu369), tryptophan‐384 (Trp384), proline‐413 (Pro413), leucine‐446 (Leu446) and leucine‐483 (Leu483). The residues Asp168 and Glu281 have been proposed to form a carboxylate–carboxylate pair, sharing a proton to allow the formation of a hydrogen bond (Malito *et al.*, 2004).

**Figure 6:**
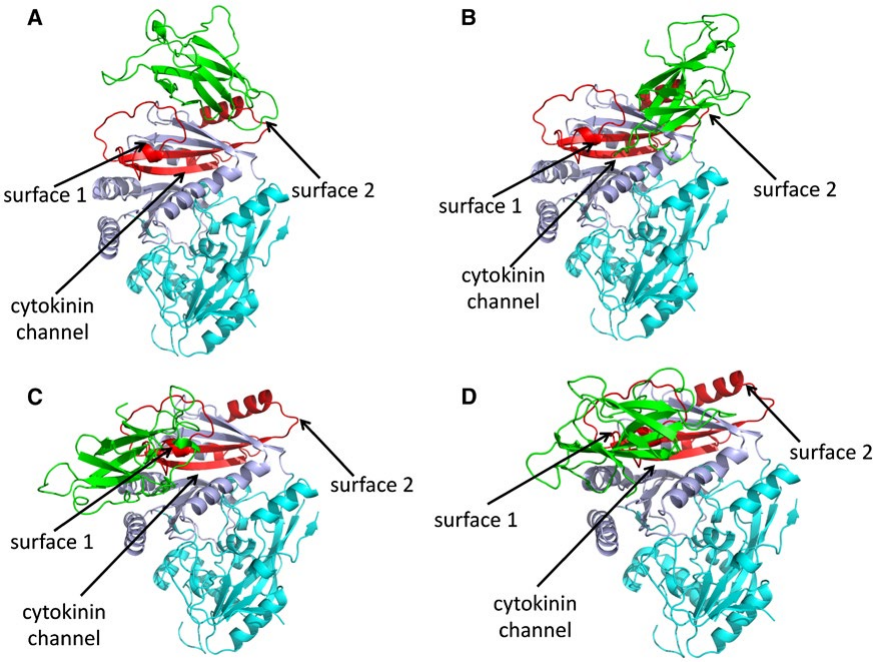
LuCKX1.1–AvrL567‐A docking studies. The structural models in (A), (B), (C) and (D) represent the top four docking models detected by GRAMM‐X for the proteins LuCKX1.1 and AvrL567‐A (green). The flavin adenine dinucleotide (FAD)‐binding and substrate‐binding domains of LuCKX1.1 are shown in cyan and light blue, respectively. The identified minimal binding region in LuCKX1.1 is coloured red. Black arrows indicate surface 1, surface 2 and the cytokinin channel. [Colour figure can be viewed at wileyonlinelibrary.com]

The iA1.1 clone initially isolated by Y2H screening encodes residues 268–340 of LuCKX1.1, indicating that these 72 residues are sufficient for the interaction with AvrL567‐A. In the crystal structure, this fragment corresponds to two buried β‐strands and two surface‐exposed regions of helices and loops (Fig. 5). One surface‐exposed region, designated hereafter as surface 1, is a loop region that links the two buried β strands. Surface 1 is right above the opening of the channel through which cytokinins enter the active site. The other surface‐exposed region, designated hereafter as surface 2, consists of short helices and loops from both ends of iA1.1. Surface 2 is further away from the cytokinin channel. Docking analysis of the LuCKX1.1 and AvrL567‐A (Wang *et al.*, 2007) structures was performed using GRAMM‐X (Tovchigrechko and Vakser, 2006), and four of the top ten models contained surface 1 in the interaction interface (Fig. 6), further suggesting the involvement of surface 1 in this interaction. A recent study on the structures of several ZmCKX isoforms (Kopecny *et al.*, 2016) has suggested that a region corresponding to surface 1 in ZmCKX could potentially be involved in the determination of substrate specificity and activity. Thus, the binding of Avr567‐A to surface 1 could influence LuCKX1.1 enzymatic activity by altering substrate access to the active site.

### Transgenic flax plants expressing *AvrL567‐A* have epinastic leaves

Given the *in vitro* effects of AvrL567‐A on the activity of LuCKX1.1, transgenic flax plants produced previously (Dodds *et al.*, 2004), which expressed AvrL567‐A under the control of the cauliflower mosaic virus 35S promoter, were re‐examined for phenotypes that might indicate hormonal changes. Seeds of several different transgenic lines segregating for the 35S‐*AvrL567‐A* transgene were planted in soil and observed during growth. The transgene co‐segregated with a leaf epinasty phenotype that was visible in cotyledons at early seedling emergence and in later leaf development up to at least 6 weeks post‐germination, but was less visible in flowering plants (Fig. S3, see Supporting Information). The transgenic plants also had a shorter stature and curling leaves compared with wild‐type plants (Fig. S3). Wild‐type and epinastic seedlings were infected with the flax rust fungus and no obvious differences were noted in disease development. The levels of several cytokinins were examined in total aboveground plant tissue at 2 days and 3 and 6 weeks post‐germination, but no clear or consistent differences were detected. The *in vivo* cytokinin measurements of wild‐type and 35S‐*AvrL567‐A* transgenic flax plants at 6 weeks post‐germination are shown in Table S2 (see Supporting Information).

## Discussion

### Avirulence effector AvrL567‐A interacts with LuCKX1.1

The *M. lini* *AvrL567* gene encodes a small protein of 127 amino acid residues that is secreted by the fungus during infection and is recognized by the flax resistance proteins L5, L6 and L7. It is unlikely that LuCKX1.1 is involved in the recognition of AvrL567‐A by the corresponding flax resistance proteins, as L5 and L6 were observed to interact directly with AvrL567‐A with appropriate recognition specificities in the absence of LuCKX1.1 (Dodds *et al*., 2006; Wang *et al.*, 2007). We did not detect any interaction between LuCKX1.1 and L6 in yeast (data not shown). The amino acid sequence and crystal structure of AvrL567‐A provide no clear indication of its virulence function (Wang *et al.*, 2007). The expression of AvrL567‐A is not essential for flax rust disease, as silencing of the *AvrL567‐A* gene had no effect on pathogen virulence (Lawrence *et al.*, 2010), possibly because of the redundant or incremental activities within the full effector complement. To provide insights into its function, a Y2H screen was used to identify proteins that interact with AvrL567‐A. One host‐derived interactor, LuCKX1, is an orthologue of Arabidopsis cytosolic cytokinin oxidase AtCKX7 and shows activity towards the cytokinins 2iP and tZ *in vitro*. These enzymes are involved in the irreversible inactivation of plant hormones in the cytokinin family. Arabidopsis, for example, produces seven cytokinin oxidases that are secreted to the apoplast or vacuole, and a single member, AtCKX‐7, which is localized to the cytosol. A cytosolic location is appropriate for an interaction with the AvrL567 protein after its translocation into host cells.

### *In vitro* and structural analysis of the interaction of AvrL567‐A with LuCKX1.1

Although interaction between AvrL567‐A and LuCKX1.1 was detected in yeast and *in planta*, a direct interaction *in vitro* could not be detected using the purified proteins. Nevertheless, the presence of AvrL567‐A enhanced the *in vitro *enzymatic activity (*K*_cat_) of LuCKX1.1 towards the cytokinins 2iP and tZ in kinetic assays by 50% and 130%, respectively.

The LuCKX1.1 crystal structure is similar to that known for other CKX enzymes with a two‐domain topology (Bae *et al.*, 2008; Kopecny *et al.*, 2016; Malito *et al.*, 2004). A 72‐amino‐acid region sufficient for Y2H interaction with AvrL567 (Fig. 1) corresponds to two surface‐exposed regions in the LuCKX1.1 structure. The surface 1 region, containing a flexible loop immediately above the cytokinin channel, also represents an interacting hotspot in docking studies between LuCKC1.1 and AvrL567‐A. The regions corresponding to surface 1 in other known CKX structures vary in both sequence and structural conformation. The structures of maize CKXs show that only ZmCKX1 adopts a well‐ordered conformation in the region corresponding to surface 1 with two helices (Kopecny *et al.*, 2016; Malito *et al.*, 2004). In Arabidopsis AtCKX7, this region corresponds to two short helices with connecting loops (Bae *et al.*, 2008) and, in flax LuCKX1.1, it largely comprises a loop containing a short helix. In the high‐resolution crystal structure of LuCKX1.1, the electron density map for the surface 1 region is not well resolved, indicating flexibility in this loop region. The same region in the ZmCKX2 structure is only partially resolved as a loop similar to LuCKX1.1 and, in the ZmCKX4a structure, it is completely disordered and unresolved (Kopecny *et al.*, 2016), suggesting hypervariability and flexibility of the region. In maize, the secreted ZmCKX1 exhibits quite different substrate specificity and kinetics from other studied variants, including ZmCKX2, ZmCKX3, ZmCKX4a, ZmCKX4b and ZmCKX10 (Kopecny *et al.*, 2016; Smehilova *et al.*, 2009). The important residues in the active sites are largely conserved in these CKXs, and mutational studies of some potentially relevant residues surrounding the active sites could not explain the substrate specificity of ZmCKX1 (Kopecny *et al.*, 2016). The region delineating the substrate entrance of CKXs is relatively far away from the active site, but represents the only hypervariable region among the ZmCKX variants with crystal structures available (Kopecny *et al.*, 2016), and thus could play a potential role in substrate selection. Based on the structures of ZmCKX1 and ZmCKX4a bound to different substrates, it was proposed that the region corresponding to surface 1 may contribute to substrate specificity for N9‐substituted cytokinins by binding to the ribose cytokinin moiety (Kopecny *et al.*, 2016). ZmCKX1 is more active as an enzyme than other variants (Kopecny *et al.*, 2016; Smehilova *et al.*, 2009). Thus, AvrL567‐A binding to the flexible surface 1 region of flax LuCKX1.1 could potentially cause conformational changes in surface 1 and influence substrate access to the active site, resulting in enhanced enzymatic activity. The potential function of surface 1 in CKX substrate specificity and catalysis requires further investigation.

### Potential role of AvrL567‐A in cytokinin signalling

The levels of active cytokinin in plant cells are determined by synthesis, irreversible inactivation by cytokinin oxidases and reversible enzymatic transitions between active and inactive derivatives of the hormone. The role of cytosolic cytokinin oxidases relative to other cytokinin oxidases in the apoplast and vacuole is not well understood. Mutation of the single cytosolic gene *AtCKX7* in Arabidopsis has no visible phenotype (Kollmer *et al.*, 2014), but overexpression in transgenic Arabidopsis and rice of their cytosolic cytokinin oxidase genes alters cytokinin levels and plant morphology in terms of root growth and reduced plant height (Gao *et al.*, 2014). Although we did not detect a reduction in the level of any cytokinins in total tissue extracts from plants expressing AvrL576, this may be a result of the masking effects of the much larger pools of cytokinins in the apoplast and vacuoles compared with the cytosol (Jiskrova *et al.*, 2016). Furthermore, leaf epinasty was evident in the first true leaves exiting the apical bundle of leaves encompassing the meristem. Transgenic tobacco plants overexpressing AtCKX1 also showed epinastic leaves (Werner *et al.*, 2001). Potentially, the epinastic leaf phenotype may be caused by cytokinin changes only at or near the apical meristem, and these changes may also be masked in the analysis of total aboveground tissue.

Although cytokinins are mainly known for their control of plant cell division, growth and development, more instances of their roles in plant–pathogen interactions are appearing and this is an expanding area of study (Albrecht and Argueso, 2017; Naseem *et al.*, 2014). The involvement of increased levels of cytokinins in plant tumour and leafy gall diseases, such as crown gall and witches broom, is well known (Naseem *et al.*, 2014). More recently, involvement in other diseases has been documented. For example, the *Pseudomonas* HopQ1 effector has been reported to directly modulate plant endogenous cytokinins (Hann *et al.*, 2014). This effector possesses nucleoside hydrolase activity and up‐regulates the level of active cytokinins and suppresses the accumulation of FLAGELLIN‐SENSING 2 (FLS2) and PTI. This work also showed that low doses of exogenously applied cytokinins suppressed PTI and led to the increased susceptibility of *Nicotiana benthamiana* to a mutant of *Pseudomonas syringae *pv. *tomato *(*Pto*) DC3000 unable to secrete effectors that suppress PTI, whereas high doses of cytokinins had the opposite effect. Another study showed that increased levels of cytokinins, through exogenously supplied tZ or transgenic overexpression of isopentenyl transferase, increased the basal resistance of *A. thaliana* to *Pto* DC3000*,* by the induction of several pathogenesis‐related (PR) genes in the SA‐dependent pathway (Choi *et al.*, 2010). Conversely, Choi *et al.* (2010) also showed that transgenic plants overexpressing a CKX and host mutants that failed to perceive endogenous cytokinins showed reduced basal resistance. Constitutive activation of the NLR protein UNI in *Arabidopsis* induced PR gene expression via the accumulation of SA and tZ‐type cytokinins (Igari *et al.*, 2008). However, overexpression of *AtCKX1* inhibited the up‐regulation of PR genes in a background of constitutive UNI activation. These results strongly indicate the involvement of cytokinins, especially tZ types, in SA‐mediated defence and PR gene expression. Thus, the down‐regulation of particular cytokinins resulting in the suppression of defence responses could be a pathogen virulence strategy to suppress plant basal defence. So far, the only reported study of a pathogen down‐regulating plant endogenous cytokinins showed that the hemibiotrophic fungus *Verticillium longisporum* induced a subset of *Arabidopsis* CKX genes, leading to reduced cytokinin levels (Reusche *et al.*, 2013). However, whether this is the result of effectors secreted by the fungus has not yet been demonstrated. The emerging theme of these studies is that the effects of cytokinins in plant defence are complex, and fine‐tuning of plant cytokinin levels may be critical for pathogen colonization. Thus, we hypothesize that translocation of AvrL567‐A into the cytosol of host cells leads to increased activity of LuCKX1.1, and reduces the cytosolic cytokinin levels, thereby benefiting the pathogen.

In rust diseases, including flax rust, ‘green islands’ are often observed around infection sites as the uninfected remains of the leaf begin to naturally senesce. This effect can be mimicked by adding spots of exogenous cytokinin to senescing leaves, and so it is thought that green islands are induced in rust‐infected plants by increased cytokinin levels around infection sites. The ‘green island’ effect and the activation of cytokinin oxidase are contradictory, with the latter expected to decrease rather than increase cytokinin levels at the site of infection. It may be that increased extracellular cytokinin levels surrounding infection sites may be beneficial in causing nutrient flow from the host towards the pathogen, whereas the reduction in cytokinin levels within infected cells may be beneficial in suppressing basal immunity. Clearly, the role of cytokinin in rust disease warrants further investigation.

## Experimental Procedures

### Y2H screening

An AvrL567‐A cDNA fragment encoding amino acids 24–150 (lacking signal peptide) was cloned into the vector pGBKT7 and transformed into the yeast strain Y187 as bait. A cDNA library was generated from total RNA extracted at 6 days post‐inoculation from Hoshangabad flax plants infected with the flax rust strain CH5. mRNA was isolated from total RNA using a PolyATract mRNA isolation system (Promega, Madison, WI, USA). First‐strand cDNA construction was performed according to the manufacturer’s protocol (Clontech, Matchmaker™ Library Construction and Screening Kits, Mountain View, CA, USA). Second‐strand synthesis and amplification were performed using long‐distance polymerase chain reaction (PCR). Double‐stranded cDNA and pGADT7‐Rec were co‐transformed into competent AH109 yeast cells. A 1‐mL aliquot of the frozen cDNA library in AH109 (prey) was thawed and combined with roughly 7 × 10^8^ Y187 cells containing the bait plasmid pGBKT7 AvrL567‐A and resuspended in a total volume of 50 mL in 2 × YPDA (10 g bacto yeast extract, 20 g bacto peptone, 20 g glucose monohydrate, and 40 mg adenine hemisulfate in 1 L water) plus 50 μg/mL kanamycin in a 1.5‐L flask. The cells were incubated for 20 h at 30 °C with gentle swirling (30 rpm). When the mating was complete, and no zygotes were visible under a phase contrast microscope, the cells were spread on plates lacking tryptophan, leucine and histidine (–HTL) for the selection of positive interactions.

### BiFC assay and confocal fluorescence microscopy

Relevant LuCKX1.1, AvrL567‐A and AvrM constructs were cloned into pDest‐YFP, pDest‐YN or pDest‐YC vectors (Bernoux *et al.*, 2008) and transformed into *Agrobacterium tumefaciens* strain GV3103. Transformed *A. tumefaciens* colonies were inoculated into 50 mL of LB (10 g bacto tryptone, 5 g yeast extract, and 10 g NaCl in 1 L water) with appropriate antibiotic selection and incubated at 28 °C with shaking for 2 days. Cells were pelleted, washed with 20 mL of deionized H_2_O and resuspended in 15 mL of deionized H_2_O containing 10 mm MgCl_2_ and 200 μm acetosyringone. Samples were incubated for 2 h at room temperature and the cell suspension was diluted to an optical density at 600 nm (OD_600nm_) of 1.0 prior to infiltration. In the case of co‐infiltration with two Agrobacterium transformants containing different plasmids, a final OD_600nm_ of 0.5 was used for each construct. Two‐ to four‐week‐old flax plants (cv. Hoshangabad) were infiltrated with a 1‐mL syringe on the abaxial side of the leaves and the plants were placed in a misting chamber for 2–5 days. Leaves collected for microscopy were placed on ice until examination by confocal fluorescence microscopy within 2 h of collection. Leaves collected for western blots were immediately frozen in liquid N_2_ and stored at −80 °C until further use.

For microscopic examination, most of the leaf tissue surrounding the point of infiltration (circle of 0.4 mm in diameter) was removed and the excised disc was placed on a glass slide; a drop of water was placed on the abaxial side and the leaf sample was covered with a cover slip. Leaf discs were examined with a Leica (Buffalo Grove, IL, USA) SP2 confocal laser scanning microscope with the following settings: excitation beam splitter set at double dichroic DD 458/514; excitation wavelength, 514 nm; pinhole set to 1 airy unit; three photomultiplier tubes (PMT) active (PMT1, 525–610 nm; PMT2, 625–690 nm; PMT Trans, visible light). PMT1 was used to detect YFP fluorescence, PMT2 was set to detect background/non‐specific fluorescence and PMT Trans was set to record the bright field image of the object. Images were taken of individual horizontal segments of the flax leaf epidermal and mesophyll cells and of multiple horizontal planes by scanning through the leaf tissue from the abaxial to the adaxial side or vice versa (*z*‐stacks). Leica confocal software (LCS) was used to operate the microscope and to obtain the images, and LCS Lite was used to analyse the images and to prepare image overlays.

### Immunoblot analysis

Plant proteins were extracted by grinding two leaf discs collected 28 h after agroinfiltration in loading buffer. Proteins were separated by sodium dodecylsulfate‐polyacrylamide gel electrophoresis (SDS‐PAGE) and transferred to nitrocellulose membranes (Pall, Port Washington, NY, USA). Membranes were blocked in 5% skimmed milk and probed with 0.2 μg/mL anti‐green fluorescent protein (anti‐GFP) mouse monoclonal antibodies (Roche, Indianapolis, IN, USA), followed by 1 : 20 000 dilution of goat anti‐mouse horseradish peroxidase (HRP) conjugate (Bio‐Rad, Hercules, CA, USA). HRP activity was detected with Super Signal West Pico chemiluminescent substrate (Thermo Scientific, Waltham, MA, USA). Amersham (Waltham, MA, USA) Hyperfilm ECL was exposed to the membranes for 2–20 min depending on the signal strength and films were developed in an AGFA (Elmwood Park, NJ, USA) CP1000 automatic developer.

### Protein purification, endpoint assays and kinetic assays

The LuCKX1.1 protein (residues 44–534) was purified as described by Wan *et al.* (2013). The AvrL567‐A protein (residues 24–150) was purified as described by Wang *et al.* (2007).

In the endpoint assays, the electron acceptor DCPIP (Sigma‐Aldrich, Raleigh, NC, USA) was applied in McIlvaine buffer (100 mm citric acid and 200 mm Na_2_HPO_4_) at pH 7.5. The 600‐μL reaction mixture contained 0.5 mm DCPIP, 0.25 mm cytokinin and 1.6 μm LuCKX1.1. The reactions were incubated for 1–3 h at 37 °C and then terminated by the addition of 300 μL of 40% trichloroacetic acid (TCA) (Sigma‐Aldrich). The reaction samples were centrifuged at 12 000 ***g*** for 5 min and the supernatant was transferred to 1.5‐mL microfuge tubes. The coloured Schiff base was formed by the addition of 200 μL of 2% 4‐aminophenol (Sigma‐Aldrich) in 6% TCA; 200 μL of the final reactions were transferred to a 96‐well plate, which was immediately scanned from wavelengths 300 to 500 nm at 5‐nm intervals using a UV/VIS spectrophotometer. The absorbance readings were corrected against a blank sample without using LuCKX1.1. Concentrations of Schiff bases were calculated as: concentration = *A*/(*ε* × *d*) [*A*, absorbance; *ε*, extinction coefficient; *d*, thickness of the ‘path length’ (0.5 cm)]. *ε* values for 2iP, tZ, BA and kinetin are *ε*_352nm_ = 15.2, ε_352nm_ = 3.4, ε_380nm_ = 2.5 and ε_380nm_ = 4.0 mM^−1^ cm^−1^, respectively, as described by Frebort *et al.* (2002).

To investigate whether LuCKX1.1 degrades cZ, similar assays were performed. The 200‐μL reaction mixture in a 96‐well plate contained 0.5 mm DCPIP, 0.25 mm cytokinin and 1.6 μm LuCKX1.1 in McIlvaine buffer at pH 7.5. LuCKX1.1 was added last to the reaction and mixed for 30 s before scanning at 600 nm using a UV/VIS spectrophotometer. The absorbance readings were corrected against a blank reaction without adding LuCKX1.1.

To conduct the kinetic assays and check the effect of AvrL567‐A on LuCKX1.1 enzymatic activity, a modified assay based on the bleaching of DCPIP was performed. In the control group, the 200‐μL reaction mixture in a 96‐well plate contained 0.5 mm DCPIP, 1.6 μm LuCKX1.1 and cytokinin with concentrations ranging from 0 to 500 μm in McIlvaine buffer at pH 7.5. In the experimental group, 1.6 μm AvrL567‐A was added for the investigation of its effect on LuCKX1.1 enzymatic activity. LuCKX1.1 was added last to the reactions and mixed for 20 s before scanning at 600 nm using a UV/VIS spectrophotometer. The absorption of each sample was corrected against a blank reaction without adding LuCKX1.1. The initial velocity of the reaction was calculated based on the change in absorbance in the first 20 s.

### Structure determination of LuCKX1.1

The flax LuCKX1.1 protein (residues 44–534) was expressed, purified and crystallized as described by Wan *et al.* (2013). The structure was solved by molecular replacement using Phaser (McCoy *et al.*, 2007) with AtCKX7 (PDB ID: 2EXR) as the template. The protein crystals have the symmetry of the space group C2 and contain two LuCKX1.1 molecules per asymmetric unit. Automatic model building was carried out using AutoBuild within the Phenix software package (Adams *et al.*, 2010). The resulting model was refined using Phenix.refine (Adams *et al.*, 2010) and iterative model building between rounds of refinement was performed in Coot (Emsley and Cowtan, 2004). Structure validation was carried out using MolProbity (Chen *et al.*, 2010). The structure of LuCKX1.1 was deposited in the PDB with ID 6C80.

### Cytokinin quantification

The extraction and quantification of cytokinins from plant tissues were performed with ultra‐performance liquid chromatography (UPLC)‐tandem mass spectrometry (AQUITY UPLC™ System/XEVO‐TQS; Waters, Raleigh, NC, USA) with an ODS column (AQUITY UPLC BEH C_18_, 1.7 µm, 2.1 mm × 100 mm, Waters), as described previously (Kojima *et al.*, 2009).

## Supporting information

Fig. S1 Phylogenetic analysis of flax and Arabidopsis cytokinin oxidases. LuCKX1‐related sequences from flax were obtained by BLASTp searches of the *Linum usitatissimum* var. CDC Bethune L genome (Cloutier *et al*., 2014) and protein sequences were aligned with the Arabidopsis CKX family (AtCKX1 to AtCKX7) using ClustalW and a tree generated by PhyML (Guindon *et al.*, 2010). Bootstrap values are shown for each branch.

Fig. S2 Protein expression detection in bimolecular fluorescence complementation (BiFC) assays using immunoblotting. Immunoblot detection of AvrL567‐A, AvrM, LuCKX1.1 and LuCKX1.2 fusion proteins using relevant anti‐YFP, anti‐YN or anti‐YC antibodies. YFP, yellow fluorescent protein.

Fig. S3 The phenotype of a *35S‐AvrL567‐A *transgenic flax plant. The leaf curling and reduced plant size phenotype of an *AvrL567‐A* transgenic flax plant relative to a wild‐type (WT) flax plant at 6 weeks post‐germination.

Table S1 LuCKX1.1 structure refinement statistics.

Table S2 Cytokinin measurements in wild‐type and 35S‐*AvrL567‐A* transgenic flax plants at 6 weeks post‐germination.
