# Supplementary figures and images for: Structural and functional insights into the modulation of the activity of a flax cytokinin oxidase by flax rust effector AvrL567‐A
Source: Mol Plant Pathol. 2018 Nov 15;20(2):211–22. doi: 10.1111/mpp.12749 (PMC6637871; doi:10.1111/mpp.12749)

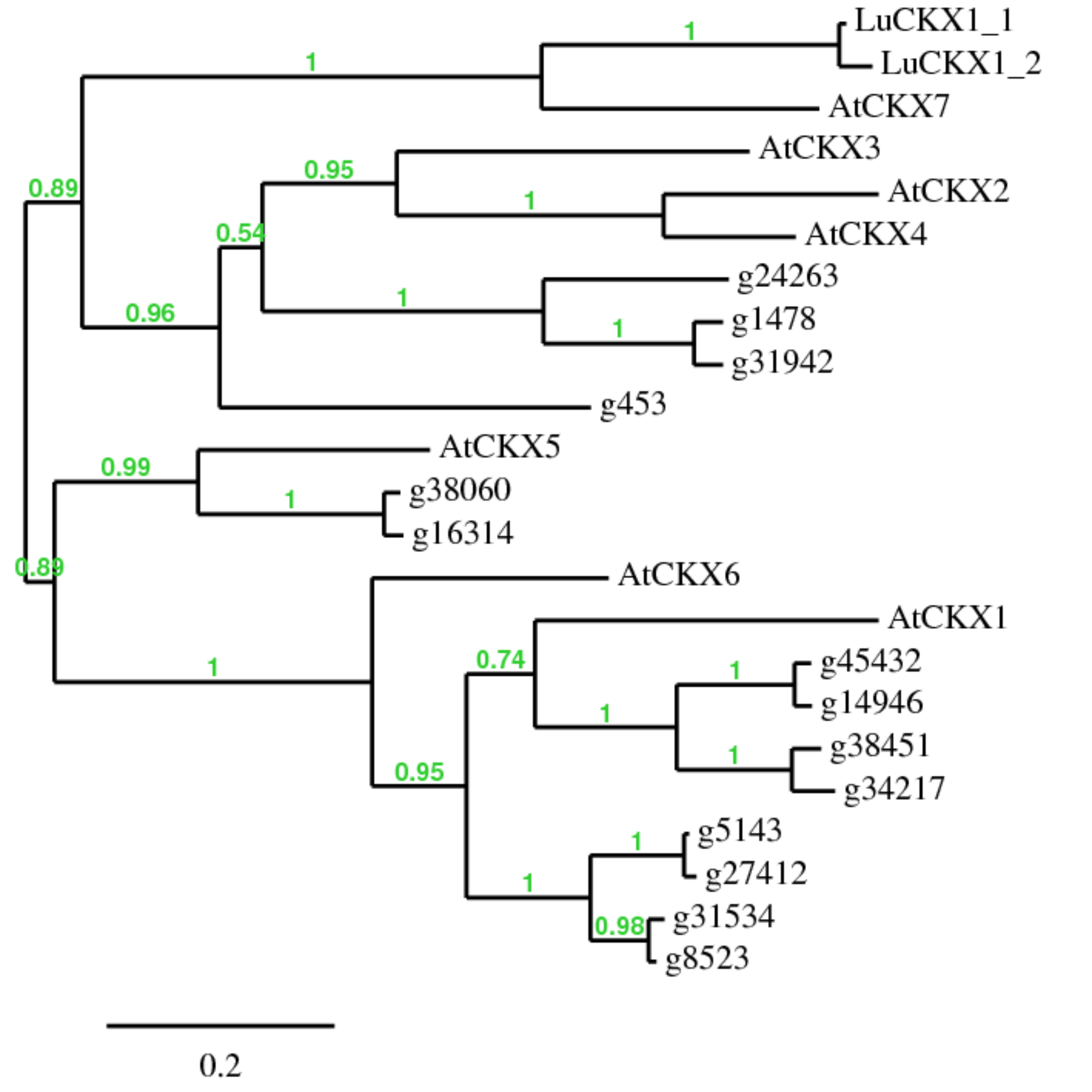

Supplement: Supplementary file 1 — Fig. S1 Phylogenetic analysis of flax and Arabidopsis cytokinin oxidases. LuCKX1‐related sequences from flax were obtained by BLASTp searches of the Linum usitatissimum var. CDC Bethune L genome (Cloutier et al., 2014) and protein sequences were aligned with the Arabidopsis CKX family (AtCKX1 to AtCKX7) using ClustalW and a tree generated by PhyML (Guindon et al., 2010). Bootstrap values are shown for each branch. [file MPP-20-211-s001.png]

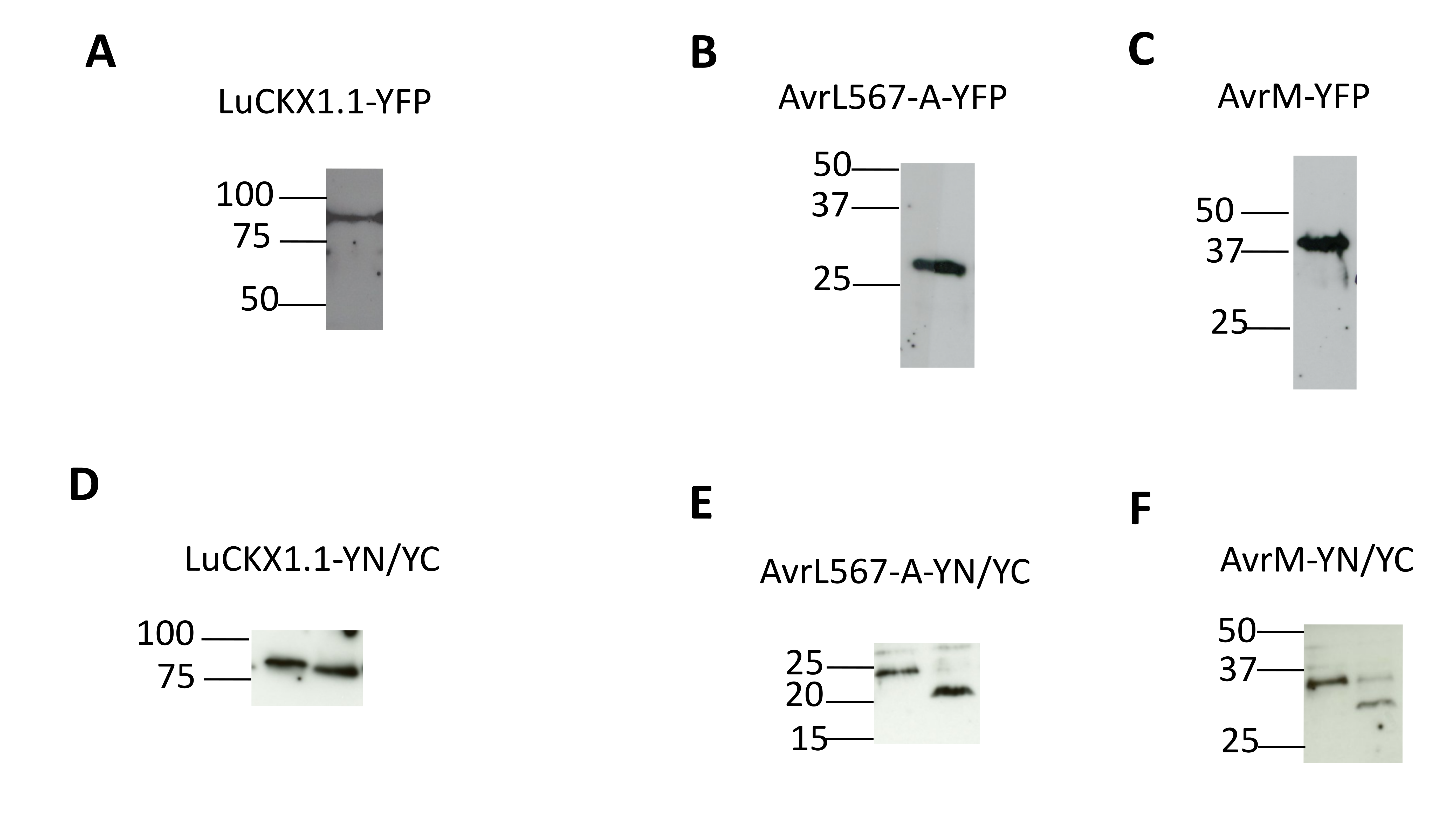

Supplement: Supplementary file 2 — Fig. S2 Protein expression detection in bimolecular fluorescence complementation (BiFC) assays using immunoblotting. Immunoblot detection of AvrL567‐A, AvrM, LuCKX1.1 and LuCKX1.2 fusion proteins using relevant anti‐YFP, anti‐YN or anti‐YC antibodies. YFP, yellow fluorescent protein. [file MPP-20-211-s002.png]

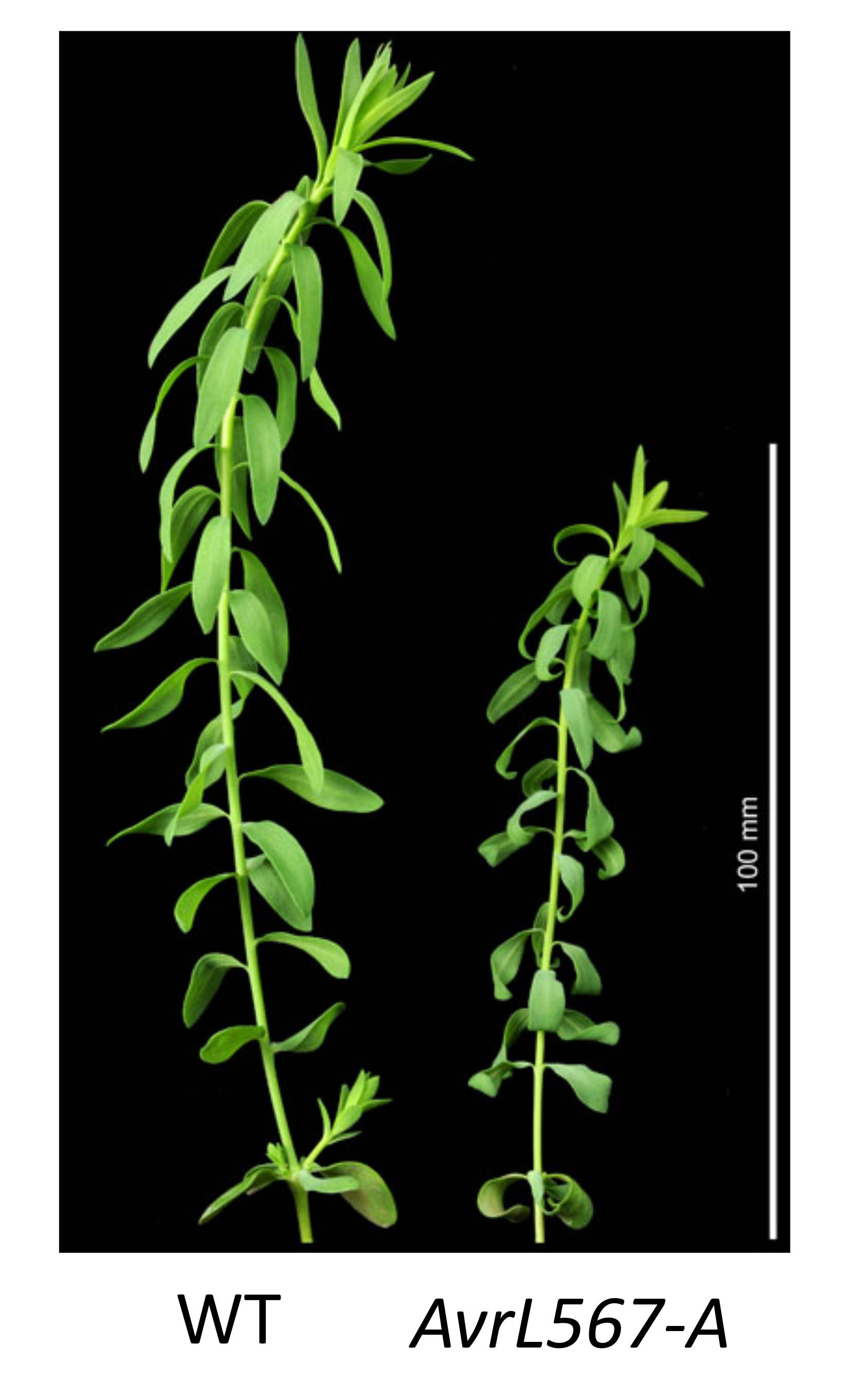

Supplement: Supplementary file 3 — Fig. S3 The phenotype of a 35S‐AvrL567‐A transgenic flax plant. The leaf curling and reduced plant size phenotype of an AvrL567‐A transgenic flax plant relative to a wild‐type (WT) flax plant at 6 weeks post‐germination. [file MPP-20-211-s003.png]
