# Supplementary material for: Structural and functional insights into the modulation of the activity of a flax cytokinin oxidase by flax rust effector AvrL567‐A
Source: Mol Plant Pathol. 2018 Nov 15;20(2):211–22. doi: 10.1111/mpp.12749 (PMC6637871; doi:10.1111/mpp.12749)
Supplement: Supplementary file 4 — Table S1 LuCKX1.1 structure refinement statistics. [file MPP-20-211-s004.pptx]

## Slide 1
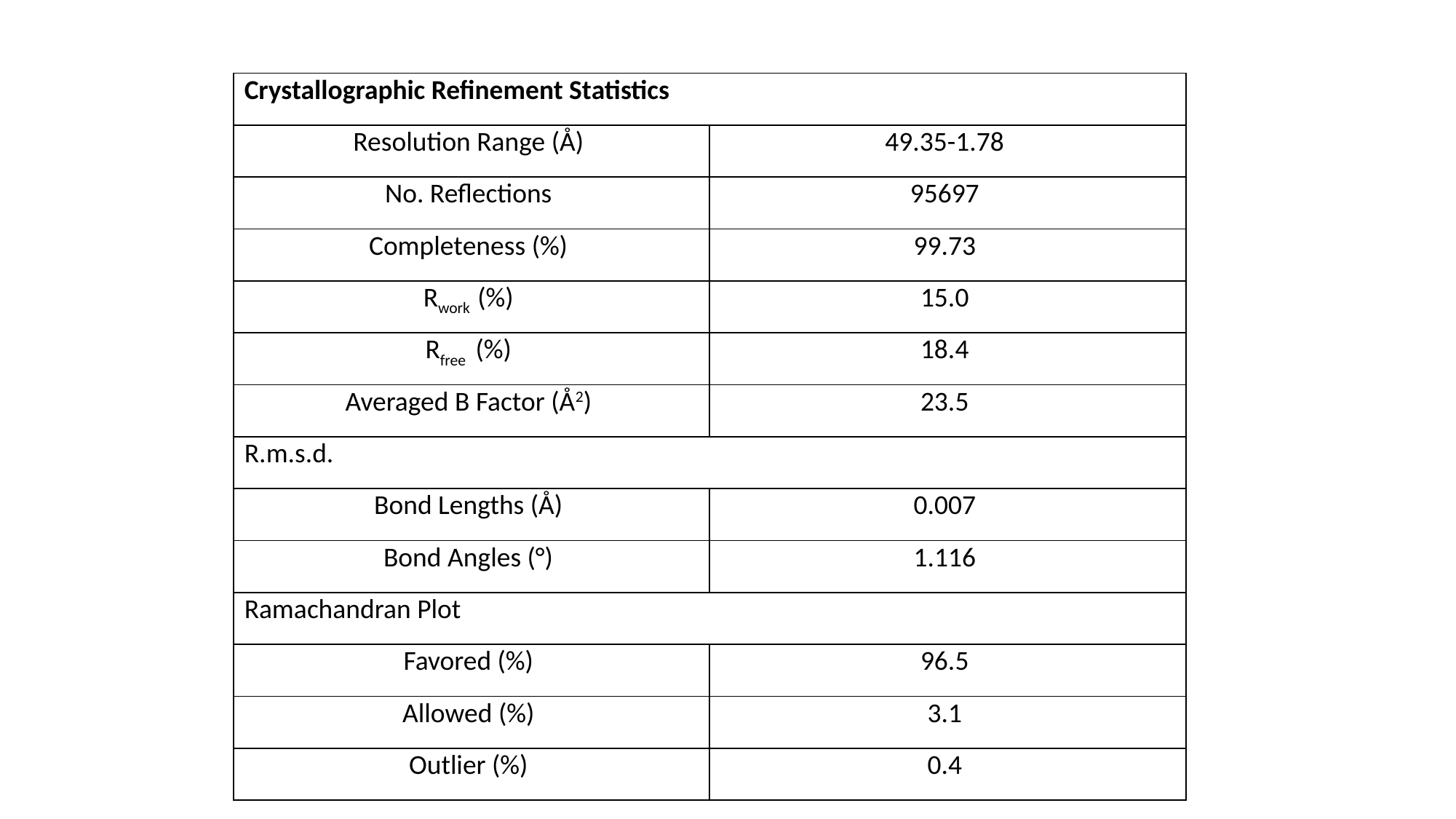

Table S1
| Crystallographic Refinement Statistics | |
| --- | --- |
| Resolution Range (Å) | 49.35-1.78 |
| No. Reflections | 95697 |
| Completeness (%) | 99.73 |
| Rwork (%) | 15.0 |
| Rfree (%) | 18.4 |
| Averaged B Factor (Å2) | 23.5 |
| R.m.s.d. | |
| Bond Lengths (Å) | 0.007 |
| Bond Angles (°) | 1.116 |
| Ramachandran Plot | |
| Favored (%) | 96.5 |
| Allowed (%) | 3.1 |
| Outlier (%) | 0.4 |
